# Incorporating genome-scale tools for studying energy homeostasis

**DOI:** 10.1186/1743-7075-3-40

**Published:** 2006-11-03

**Authors:** R Michael Raab

**Affiliations:** 1 Agrivida, Inc., Cambridge, MA, USA; 2 Department of Chemical Engineering, University of Virginia, Charlottesville, VA, USA

## Abstract

Mammals have evolved complex regulatory systems that enable them to maintain energy homeostasis despite constant environmental challenges that limit the availability of energy inputs and their composition. Biological control relies upon intricate systems composed of multiple organs and specialized cell types that regulate energy up-take, storage, and expenditure. Because these systems simultaneously perform diverse functions and are highly integrated, they are extremely difficult to understand in terms of their individual component contributions to energy homeostasis. In order to provide improved treatments and clinical options, it is important to identify the principle genetic and molecular components, as well as the systemic features of regulation. To begin, many of these features can be discovered by integrating experimental technologies with advanced methods of analysis. This review focuses on the analysis of transcriptional data derived from microarrays and how it can complement other experimental techniques to study energy homeostasis.

## Background

Mammalian control of energy homeostasis is extremely complex and integrates regulation at an organ level, cellular level, and ultimately a molecular level. In healthy humans this results in a system that matches caloric intake to energy expenditure within 0.17% during the course of a year in which approximately one million calories are consumed [1]. Understanding the genetic basis for this regulation will provide the opportunity to develop treatments for obesity and diabetes that are specifically tailored to distinct patient groups [2].

Energy homeostasis is a genetically complex and quantitative phenotype, whose molecular basis depends upon pathways involving thousands of molecules. To date, more than 600 genes, markers, and chromosomal regions have been associated or linked to obesity phenotypes [3], however, no single gene mutation can account for the variance in patient responses to a dietary treatment.

To develop a molecular understanding of mammalian energy homeostasis, the genes that underlie clinical observations must be identified. Although association studies [4], linkage studies [5], admixture studies and others that can identify quantitative trait loci (QTL, defined as any region in the genome that contributes to a quantitatively measured phenotype, such as height, weight, serum glucose levels, etc.) will continue to discover new genetic associations to weight and obesity, one complementary technique that can rapidly identify new candidate genes is transcriptional profiling. The advantage of transcriptional profiling is that it can look at thousands of genes simultaneously, and unlike mapping techniques, it looks directly at genes themselves and not just chromosomal regions.

DNA microarrays provide an efficient route to finding gene targets involved in quantitative traits and biological processes associated with complex phenotypes, such as energy homeostasis. The core concept is simple: genes that are differentially expressed between control and experimental samples may play a role in the observed differences in phenotypes. For example, C57/BL/6J mice treated with a high-fat, high calorie diet are known to become obese and insulin resistant [6,7]. Their evolving physiology is related to changes in transcription of genes mediating or responding to the treatment. Conversely, AJ mice fed the same diet are resistant to obesity and maintain glucose levels [8]. Comparing transcriptional differences between these two strains under the same conditions may help identify genes that are related to their physiology [9], if such transcriptional changes can be efficiently found and experimentally tested.

Transcriptional profiling *quantitatively *determines which genes are active or inactive in the environment from which the samples are taken. Thus, as opposed to looking for specific gene mutations that associate with energy homeostasis phenotypes (such as obesity (resistance), or insulin resistance), cellular responses from one treatment or genotype are compared with the responses from a different treatment or genotype to determine which genes are differentially expressed during phenotypic changes. This information can then be used in more detailed studies to screen for mutations and characterize relevant genes.

The advantages of using DNA microarrays for gene discovery, particularly with respect to complex diseases, are that they provide information on known or putative genes, require fewer samples than are necessary to identify quantitative trait loci (QTLs), are highly parallel, and allow direct, hypothesis based testing on a genomic scale. The fact that microarrays can directly implicate specific genes is a considerable advantage given the sample size required for QTL analysis, which only identifies genomic regions. So long as the variance in the array measurements can be quantified, direct statistical comparisons of transcript levels can be made with a moderate number of replicates.

The caveats of using DNA microarrays are that changes in gene transcription alone may not be responsible for phenotypic changes, and analysis can be challenging when confronted by 20,000 different transcript measurements. It is often wrongly inferred that changes in transcript levels correlate to changes in protein levels, or even worse, changes in protein activity, which is not true in many cases [10,11]. While increases or decreases in transcription *may *alter protein levels, there is no single correlation or function that tells how the concentration of mRNA is linked to the concentration of protein. Since it is often accepted that most phenotypes are the results of protein activity, measuring transcript levels alone will not necessarily define the genes underlying a given phenotype and other data is often required.

Incorporating DNA microarrays and other genome-scale technologies in studies of energy homeostasis promises to provide information that will more thoroughly define important molecular pathways. Despite the potential of DNA microarrays, there are several challenges that researchers often confront when beginning to use this technology in their studies. The first is which system to use given the multitude of existing systems and possible differences. The second is how to extract the most relevant information, when confronted with perhaps hundreds of differentially expressed genes. The third is how to effectively integrate other data so that the relevance of an observed change in expression can be evaluated with respect to the phenomena of interest. Each of these topics will be discussed in this review.

## Microarray systems and data acquisition

DNA microarrays rely upon labeling mRNA populations and then rapidly separating them on the array to generate signals that can be quantitatively compared. The first attempts at transcription monitoring were rather modest: Patrick Brown's initial report [12] measured the transcript levels of only 45 genes simultaneously on one array. Today, arrays containing more than 20,000 gene probes are not uncommon [13,14].

There are currently two DNA microarray technologies that are most commonly used for monitoring transcription. One is a high density oligonucleotide system commercially available through Affymetrix (Santa Clara, CA), the other is typically referred to as a "cDNA system." While there are substantial differences between the two types of technologies [15], both quantify the distribution of transcripts from a pool of RNA.

**Figure 1:**
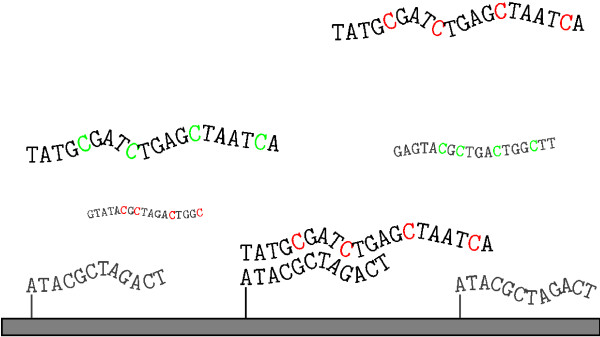
DNA microarrays work by exploiting the specificity of DNA base pairing. The initial rules for hybridization were discovered by Erwin Chargaff and dictate that each guanine noncovalently pairs with a cytosine and each adenine is paired with a thymine [92]. The affinity and stability of the hybridized, double stranded DNA is therefore directly related to sequence complementarity. In this figure the labeled "target" molecules, representing the mRNA transcripts, compete for binding to their *complementary *"probe" molecules immobilized on the array. Once equilibrium is achieved, the arrays are washed and scanned to measure the transcript abundance.

Although Affymetrix style arrays are becoming standard, "spotted" microarrays are common in academia because they provide flexibility in both the array design, and the range of assays that can be conducted. On spotted arrays, mRNA is linearly converted into a labeled cDNA, which binds to its complementary probe during hybridization, and then is quantified by measuring the label abundance as shown in Figure 1. Standardization of the experimental protocols used in RNA harvesting, purification, labeling, and array hybridization, washing, and printing (when using cDNA arrays) is critical to obtaining good data. Numerous papers have been published on these topics [12,16-19] and there are a variety of on-line resources to help experimentalists optimize their protocols [20-24].

Once the experimental protocols have been developed for a given system, reliable data can be obtained that quantitatively compares transcription levels for a vast number of genes. This data usually comes in one of two forms: a normalized intensity signal or a normalized ratio of signals.

Normalized signal intensities represent the absolute amount of labeled RNA bound to an individual gene probe of a specific sequence. Signals are usually normalized for local background fluorescence, amount of RNA in the sample, dye and labeling differences, and potentially for array to array variance. Normalized signal ratios are usually defined as the signal on one array (or in one fluorescence channel when a two-dye spotted array is used) divided by the signal on another array (or in the other fluorescence channel). In normalized ratios, one signal is typically the control signal while the other is the signal from an experimental treatment; thus a ratio of two might represent twice as much mRNA for a gene in the experimental sample compared to the control sample, while a corresponding ratio of 0.5 would represent twice as much mRNA in the control as in the experimental samples.

Ratios provide a slight problem in data analysis because the variance in values for repressed genes (that is, genes whose treatment signals are less than the control signals) is bounded between zero and one, while values for overexpressed genes are not bounded and could in principle be any number greater than unity. To overcome this issue researchers usually transform ratio data to a base two logarithmic scale (log_2_) such that a ratio of two would give a log_2 _value of +1, while a ratio of 0.5 would give a log_2 _value of -1, thereby placing both domains in a range that is amenable to linear data analysis techniques.

Data obtained are often validated using complementary techniques for a subset of probes on the array. One commonly used technique to validate transcriptional differences in a statistically meaningful way is RT-PCR, for which many varieties are available (SYBR-green based, TaqMan, etc.). This requires additional equipment, however, in recent years a number of companies have begun to offer lower cost instruments making RT-PCR validation amenable for many laboratories.

## Experimental design and data analysis

In studying energy homeostasis, valuable information can be extracted from microarray data by using statistical and data mining methods. Statistical methods rigorously quantify the reliability of differences in the microarray data [25] and can objectively evaluate changes in gene transcription ratios and derivative quantities [26]. Data mining is particularly useful for uncovering patterns and structure in microarray data that might have otherwise been difficult to detect through manual inspection and intuition [27,28]. Applying statistics and data mining methods to microarray data in unison enables rapid and reliable analysis without *a priori *assumptions that may bias the conclusions.

Selection of a particular analysis method depends largely on the experimental design and hypothesis being investigated. In all cases, proper statistical rigor should be employed, however, the significance level, correction for multiple comparisons, and other parameters can be used to arbitrarily increase or decrease the number of genes identified as having a change in transcription. While these parameters are rigorous values whose selection should be explained in any investigation, they provide some level of flexibility in selecting an overall gene set to be used subsequently in data mining.

### Statistics

Many statistical methods have been used to analyze gene transcription data [29-32]. Selection of any particular method is highly dependent upon the experimental design and type of microarray technology used.

To assess differential gene expression, a gene by gene t-test [33-35] can be applied to evaluate statistically significant expression differences in pairwise comparisons between the control and experimental samples. A common question that arises pertains to whether the Bonferroni-correction for multiple tests is appropriate [36]. Employing this correction factor will decrease the number of false positives in the data set by dramatically increasing the acceptable threshold for significance, however, it also exaggerates the number of false negatives [25], which defeats the principle advantage of using microarrays: conducting many comparisons simultaneously in parallel. One way to get around this is to employ more replicates or to validate changes of interest using a complementary method, such as RT-PCR or Northern Analysis.

Another useful method is Wilks-*λ *based ranking [37-39]. This technique is particularly appropriate for *multi-class *comparisons, ranking genes on the basis of their within group, and between group variances. Thus, a gene exhibiting a small variation within each of several groups, but large variation between groups would rank highly; conversely a gene that had a high level of variation within a group, and a low level of variation among the groups would be ranked low. The Wilks-*λ *score can be transformed into an *F *statistic, which is compared with the *F *distribution to assess the statistical significance of the observation [38].

### Data mining

There are three general types of data mining analyses commonly used with microarrays:

• *Sample Classification*: In static experiments where samples are treated with different conditions (such as diets), genes that can classify the treatments may be important in the underlying biology and therefore interesting candidates for further studies.

• *Clustering*: In experiments where each sample represents either a timepoint or a single treatment, patterns in gene transcription are observed and genes demonstrating similar responses may be co-regulated, which can lead to identifying regulatory sequences or molecular factors.

• *Systems Identification*: In experiments where it is desired to discover other kinds of interactions, including putative cause-effect relationships and relationships among different data types, these methods can be used to create models that define statistically significant relationships, whose features can be tested experimentally.

#### Sample classification

As opposed to statistical techniques that focus on the mean and variance of one variable, or differences in pairwise comparisons, multivariate techniques focus on covariances or correlations [37,39]. These methods attempt to uncover structure in the data set and identify what are the most important variables. In analyzing transcriptional data, multivariate techniques provide a way of quickly classifying treatments based upon the gene expression. For example, hypothalamic gene expression could be compared among mice fed isocaloric diets composed of normal chow, high-carbohydrate, high-fat, and high-protein. Changes in gene transcription that best predict the different treatments could then be used to classify the samples and the underlying genes would be good candidates for genotyping and additional studies. There are many different methods, however, Fisher Discriminant Analysis (FD Analysis) and Principle Component Analysis (PCA) are commonly used.

Fisher Discriminant Analysis [39-41] (FD Analysis) is a method that determines combinations of genes capable of correctly classifying the experimental samples. Thus if RNA samples were taken from normal mice, diabetic mice, and diabetic mice treated with a thiazolidinedione, FD Analysis could be used to find genes whose expression classifies these mice according to their collective gene transcription profiles. In this regard, FD Analysis is considered a *supervised *data analysis method because the sample classes are defined at the outset. FD Analysis identifies genes that best place the samples into predefined treatment classes by maximizing the distance between the classes.

FD Analysis provides *linear combinations of gene expressions *that are selected according to the discriminatory power of gene groups as opposed to individual genes. Samples are scored based on the weighted contributions of each gene to a newly defined metric called a canonical variable. Because each gene's contribution to a sample's score is weighted by a coefficient called a "loading," genes with very small loadings do not significantly contribute to the sample's score and classification, and can therefore be eliminated from further consideration. A score is thus defined as

*S *= ∑ *λ*_1_*g*_1 _+ *λ*_2_*g*_2 _+ ... + *λ*_*i*_*g*_*i *_+ ... + *λ*_*n*_*g*_*n *_    (1)

where *S *is the sample score, *λ*_*i *_represents a gene's loading, *g*_*i *_represents a gene transcription level (or ratio), and the sum occurs over all discriminatory genes, *n*.

This technique can be used as a tool to visualize microarray results in a lower dimensional space defined by the canonical variables. The canonical variables are metrics calculated as a weighted linear sum of the other variables, in this case gene expressions, as shown in Equation 1. The underlying principle is that if the scores accurately classify the samples, then the genes selected to determine the scores differentiate the treatments when sample classification is used as a criterion.

In FD Analysis the canonical variables, **V**, are selected so as to maximize class separation [40]. These variables are determined as the eigenvectors of the inter-group variance, **B**, scaled by the intra-group variance, **W**, as

**W^-1 ^BV **= **VΛ **    (2)

where

**B **= **T **- **W **    (3)

**T **= (**X **- **1**X¯T
 MathType@MTEF@5@5@+=feaafiart1ev1aaatCvAUfKttLearuWrP9MDH5MBPbIqV92AaeXatLxBI9gBaebbnrfifHhDYfgasaacH8akY=wiFfYdH8Gipec8Eeeu0xXdbba9frFj0=OqFfea0dXdd9vqai=hGuQ8kuc9pgc9s8qqaq=dirpe0xb9q8qiLsFr0=vr0=vr0dc8meaabaqaciaacaGaaeqabaqabeGadaaakeaacuWGybawgaqeamaaCaaaleqabaGaemivaqfaaaaa@2F5B@)^*T *^(**X **- **1**X¯T
 MathType@MTEF@5@5@+=feaafiart1ev1aaatCvAUfKttLearuWrP9MDH5MBPbIqV92AaeXatLxBI9gBaebbnrfifHhDYfgasaacH8akY=wiFfYdH8Gipec8Eeeu0xXdbba9frFj0=OqFfea0dXdd9vqai=hGuQ8kuc9pgc9s8qqaq=dirpe0xb9q8qiLsFr0=vr0=vr0dc8meaabaqaciaacaGaaeqabaqabeGadaaakeaacuWGybawgaqeamaaCaaaleqabaGaemivaqfaaaaa@2F5B@)     (4)

W=∑(Xj−1X¯jT)T(Xj−1X¯jT)     (5)
 MathType@MTEF@5@5@+=feaafiart1ev1aaatCvAUfKttLearuWrP9MDH5MBPbIqV92AaeXatLxBI9gBaebbnrfifHhDYfgasaacH8akY=wiFfYdH8Gipec8Eeeu0xXdbba9frFj0=OqFfea0dXdd9vqai=hGuQ8kuc9pgc9s8qqaq=dirpe0xb9q8qiLsFr0=vr0=vr0dc8meaabaqaciaacaGaaeqabaqabeGadaaakeaaieqacqWFxbWvcqGH9aqpdaaeabqaaiabcIcaOiab=HfaynaaBaaaleaacqWGQbGAaeqaaOGaeyOeI0Iae8xmaeJafmiwaGLbaebadaqhaaWcbaGaemOAaOgabaGaemivaqfaaOGaeiykaKYaaWbaaSqabeaacqWGubavaaGccqGGOaakcqWFybawdaWgaaWcbaGaemOAaOgabeaakiabgkHiTiab=fdaXiqbdIfayzaaraWaa0baaSqaaiabdQgaQbqaaiabdsfaubaakiabcMcaPiaaxMaacaWLjaWaaeWaaeaacqaI1aqnaiaawIcacaGLPaaaaSqabeqaniabggHiLdaaaa@4AF8@

and the sum occurs over all of the sample classes. In this formulation **X **represents the (*n *samples (rows) × *g *genes (columns)) data matrix, **T **represents the total variation among all the data, and the eigenvalues, Λ, indicate the discriminatory power of the canonical variables.

Sample classification is often tested by dividing the samples into training and test sets to determine the statistical significance of the findings. In these procedures, a subset of the samples can be used as a training set to develop a model that predicts the membership or other (test) samples. The membership of the training and test sets can be varied in iterations of the analysis to determine the error rate based upon false classification. Genes with large absolute values for their loadings, which are most commonly identified in successful classification models, become lead candidates.

One way to think intuitively about eigenvectors is that they represent the "factors" (variables; the genes in microarray data) that describe (that is, can be used to quantitatively predict most of) the data matrix. An eigenvector's representation is based upon how it weights a variable or "factor" within the data; variables with large absolute values in the eigenvector are important and those with values close to zero can be discarded.

A nutritional analogy would be a data set that described the caloric content of different meals based on their composition. Here, each sample would contain data on a different meal. This data would be defined by the number of calories contained in each dietary component of the meal (such as starch, cellulose, glucose, sucrose, lipids, cholesterol, protein, etc., and are analogous to genes in microarray data), which would define a sample vector such as:

Meal_1 _= [Starch, 10] + [Lipid, 2] + [Protein, 3] + ...     (6)

If the data set contained vectors for: ten meals that were analyzed and considered high in carbohydrate, another ten representing meals high in fat, and a third ten representing control meals, then FD Analysis could be used to accurately classify these meals into the predefined groups based upon the caloric content of their components. In this example the original data matrix, **X**, would have 30 rows, one for each meal, and several dietary components, *g*, whose caloric content was measured in each meal (*30 *meals × *g *dietary components). The eigenvectors selected could be used to score each meal according to the caloric content of its components, and it would be anticipated that the components, or "factors", that were most heavily weighted would be specific carbohydrate and fat components. By looking at the absolute value of the loadings for each component, one can determine which components are critical to classification of the meals into the high-carbohydrate, high-fat, and control meal groups. Furthermore, knowing the quantitative relationship between components allows a researcher to explore new hypotheses about the system: does decreasing glucose content and increasing protein content change a high-carbohydrate meal into a control meal, or can new meals be designed that lie between the groups in the FD Analysis space, and what are their properties? The gene data can be similarly analyzed and viewed in terms of genetic contribution to complex phenotypes.

**Figure 2:**
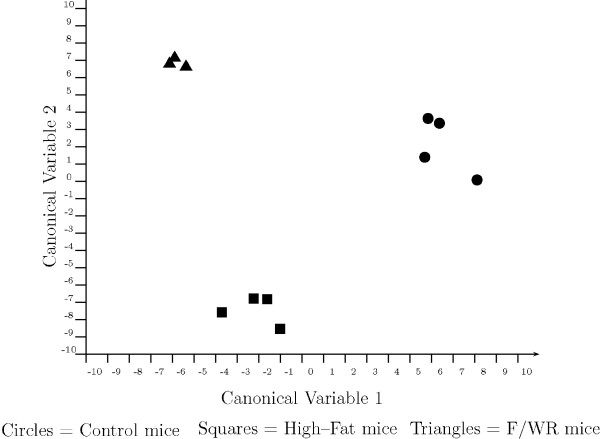
Fisher discriminant analysis plot of mouse liver samples. Samples were scored according to the canonical variables determined by Fisher Discriminant Analysis (FD Analysis). Each canonical variable is defined as a weighted sum of 100 specific genes. To score a sample, the gene expression value is multiplied by an FD Analysis coefficient, called a loading, and the products from the 100 genes used in the analysis are summed to give the canonical variable score for the sample. F/WR: Fasting/Weight Reduced.

We used FD Analysis to investigate hepatic gene regulation in response to diet induced obesity and insulin resistance [42]. In those studies whole genome microarrays containing 17,280 gene probes were used to examine transcription in three groups of C57/BL/6J mice: 1) the "control group" received a normal diet for 10 weeks, 2) the "high-fat group" received a high-fat diet for 10 weeks, and 3) the "fasted/weight reduced group", which was fed the same high-fat diet for ten weeks followed immediately by 48 hours of caloric restriction, returning their weights to baseline levels prior to tissue harvest. The resulting classification among these treatments is shown in Figure 2.

Principle component analysis (PCA) is similar to FD Analysis in that it can be used as a data reduction technique and to find structure in a data matrix. It is a multivariate classification method that, like FD Analysis, scores samples according to linear combinations of gene expressions. The difference between FD Analysis and PCA is how they choose which genes are used in the scoring procedure. For this reason both techniques can be used to find different sets of genes, some of which will be commonly identified using both algorithms.

PCA reduces the original set of variables (in this case genes) into a smaller, orthogonal set of variables that is composed of linear combinations of gene expression data, called principle components. It is the principle components that define the sample scores in the same manner as the FD Analysis canonical variables. Unlike FD Analysis, PCA is *unsupervised*, that is, it does not assign the samples to a specific class *a priori*. Instead the coordinates of the smaller, orthogonal variable set are chosen such that they capture as much of the total variance as possible in the original data. In this way, it may be possible to identify groups of genes or samples that show similar behavior.

The procedure for using PCA has been described previously [37,43,44] and the mathematics is briefly reviewed here. For a given data matrix composed of *n *samples and *g *genes, the data may be scaled and is usually transformed into a covariance or correlation matrix. The principle components are identified as the set of vectors, each containing coefficients, y_1_, y_2_, ..., y_*i*_, ..., y_*m*-1_, y_*m*_, such that y^*T *^**X **is maximized over all linear combinations of **X **with the constraint y^*T *^y = 1 for all vectors. To find this set of vectors, it has been shown that they must satisfy *g *simultaneous equations of the form

(**C **- *λ*_*i*_**I**)*y*_*i *_= 0     (7)

where **C **represents the correlation or covariance matrix, depending upon which transformation was used to convert the original data matrix.

This is the common eigenvalue, eigenvector problem. Nontrivial solutions for the eigenvectors, y_*i*_, can be found by solving for the eigenvalues, *λ*_*i*_, of the determinant

|**C **- *λ*_*i*_**I**| = **0 **    (8)

The determinant of these equations results in a polynomial of order *g*; hence the *g *roots associated with the polynomial are the eigenvalues. From this set, the first principle component can be identified by choosing the largest eigenvalue (root of the polynomial) and then solving for the corresponding eigenvector. This eigenvector gives the coefficients of the variables, genes in this case, of the first principle component. The procedure is then repeated for each of the subsequent *g *eigenvectors with the constraint that the principle components must be mutually orthogonal. Other methods of calculating the principle components are possible such as orthogonal decomposition of the input matrix or by using nonlinear iterative partial least squares [45,46].

Because PCA is not scale invariant, using either the covariance or correlation matrix will affect the solution obtained, and the resulting solutions from the two different matrix transformations will not be related. For this reason it's prudent to conduct both transformations and run the analyses in parallel.

#### Cluster analysis

Cluster analysis is used to find genes that are potentially co-regulated. The concept is simple: if one gene is induced or repressed in the same manner as another gene, across many samples (either conditions or timepoints), then the two genes may share similar regulation. While the biological significance of such a relation still must be assessed, cluster analysis provides targets for the discovery of new transcriptional regulatory elements, factors, and mechanisms.

There are numerous clustering algorithms [47-49], all of which generally follow this procedure: 1) Data normalization, 2) Data filtering, 3) Data clustering. Data normalization is used to correct for artifacts that may influence the data, such as differing dye incorporation rates, and has been reviewed substantially in the literature [26,50,51]. The most commonly used normalization methods are mean-centering and autoscaling. Mean centering reduces the mean transcriptional value of any gene across all samples to zero by subtracting the gene's mean transcriptional value from each sample value (across all samples in the data set). This causes the clustering algorithm to focus on the variance in each gene about its mean as opposed to the absolute level of transcription for any given gene. Autoscaling transforms the data into a set that is mean centered and has unit variance. This helps identify established patterns that are independent of the mean and are well conserved across the samples. Data filtering is usually used to remove noise in the data set. Many different types of filters exist and the choice of any given filter depends partially on the experimental design. It is common to remove genes that either do not have reliable values across all samples, or genes that were not statistically different in a minimum number of samples. Once the data is processed, clustering can begin.

There are many different clustering algorithms, such as K-means [52,53], nearest neighbor [54], self organizing maps [55], and hierarchial [56]. These algorithms assemble genes into groups that have similar patterns and therefore may be related. While they often will produce similar results, there are nuances to each method that can cause differences to arise and thus using multiple algorithms with a single data set may be worthwhile. For example the degree of statistical correlation between gene transcription profiles may be used as a criteria for clustering and changing the required statistical cut-off for correlation can vastly affect group membership.

Clustering can also be used to look for specific patterns of gene expression that correlate with a predefined molecular phenotype. Because the transcription data is usually normalized, mean centered or autoscaled, and unitless when it enters a clustering algorithm, other data types can easily be incorporated if they are similarly processed. Thus if the effect of a dietary treatment on adipose gene transcription was being studied, and intracellular protein levels were also measured for a set of specific proteins (via Western Analysis, mass spectrometry, or IR-fluorescence), the protein data could also be included in the data matrix. In this case genes that were in clusters correlated or anti-correlated with the proteins may be related. While the role of the genes within a cluster must be subsequently resolved, the ability of clustering to examine the relationships between genes and other physiological data is an important tool for future studies.

For example, we studied the effect glutamine concentration on hepatoma metabolism. It has been previously reported that glutamine affects glucose up-take and glycolytic flux [57,58], and can serve as a carbon source for gluconeogenesis [59] and *de novo *lipogenesis [60]. In our experiments, the concentration of glutamine was oscillated in the cells' medium causing changes in gene transcription and glycolytic flux.

**Figure 3:**
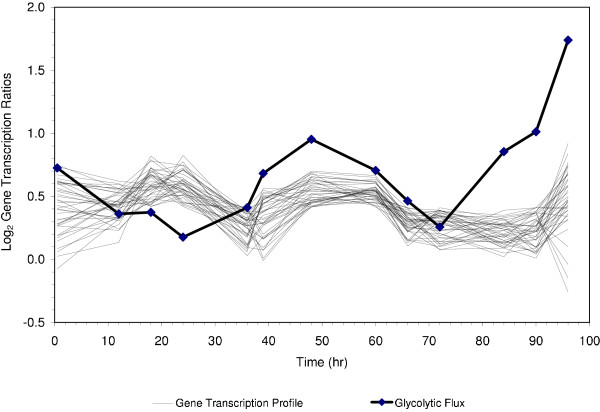
Clustering of genes related to glycolytic flux from the Teiresias algorithm. In this case Teire-sias was asked to find patterns of the type ". . -F -F -F F F F F F . . F", where "." represents any transcript value, "F" represents a transcript level that correlates with the flux, and "-F" represents a transcript level that is anticorrelated with the flux.

To identify genes that were either correlated or anti-correlated with the flux measurement, we used Pearson correlation [27] and Teiresias [49], which is a pattern discovery algorithm. Teiresias converts the expression data into discrete patterns by categorizing each transcription value into one of several predefined bins. It then finds patterns in the discretized profiles. Unlike other clustering algorithms, Teiresias searches transcriptional data for all possible patterns defined by several input parameters, including patterns that are not "full." For example, if the gene expression data is discretized into bins defined as increased (I), unchanged (U), and decreased (D) expression, then for an expression profile with five samples, Teiresias can find full patterns (such as "U D U I U") or partially full patterns (such as "(U, I) D (D, U) I U" or "U . U I ." where either value is permissable within the parentheses, and the period allows any value, I, U or D.). Figure 3 shows the result of using Teiresias to cluster genes based on their relation to the glycolytic flux determined in the experiment.

Clustering results in our hepatoma investigations showed that increased transcription of some genes was required to allow cells to respond to the changing glutamine concentration. Most of the genes found to be correlated or anti-correlated with flux were not known to be directly connected to intermediary metabolism, thus highlighting other genes and systems that are perturbed as a result of glutamine changes in the medium.

#### Systems identification

Clustering and sample classification can detect genes that are similarly expressed, whose expression levels match a pattern of interest, or genes that can classify experimental samples, however, they cannot easily relate gene transcription to quantitative metrics that describe energy homeostasis. Thus, some method of analysis is required to link identified genes to environmental perturbations or measurable changes in energy up-take, expenditure, and storage. Because both microarray and physiological data can possess many dimensions, regression methods that reduce the dimensionality of these data sets and find correlations between them are very important to integrating microarray data with other data types. Methods of analysis that can link expression data to other phenotypic markers, or that can incorporate other types of data, provide tools for the investigation of system properties [49,61-63].

One way to investigate these types of multivariate problems, where it is desired to correlate multiple inputs, represented by an "X-Block," (**X**), with multiple outputs, represented by a "Y-Block," (**Y**), is to use a regression method called partial least squares (PLS) [64]. PLS considers the *collective contributions *of the inputs to the outputs, and thus utilizes multidimensional data as opposed to other regression techniques that use data with a single dimension. It is advantageous for large systems because both **X **and **Y **are decomposed into a lower dimensional space where their relationship is explored.

As an example, we explored the application of PLS to microarray data by investigating how a murine hepatoma cell line (Hepa1-6 cells) alters its gene expression to control glycolytic flux (unpublished data). In these experiments [49], total RNA was isolated at each time point and the microarray data was used for **X**; at the same time the forward flux through phosphohexose isomerase was measured using tritiated glucose (which generates labeled water) and used for **Y**. Based on the experimental results a PLS model was created, where the transcription data (11 samples × 3,185 genes) was related to the flux measurements (11 samples × 1 flux measurement).

After autoscaling the data matrices, PLS was run to construct the model. PLS decomposes the original data matrices into a lower dimensional space and then builds a correlation between the reduced matrices. The decomposition of the original matrices is defined by their "outer" relations, given by:

**X **= **T P**^*T *^+ **E **= ∑t¯hp¯hT
 MathType@MTEF@5@5@+=feaafiart1ev1aaatCvAUfKttLearuWrP9MDH5MBPbIqV92AaeXatLxBI9gBaebbnrfifHhDYfgasaacH8akY=wiFfYdH8Gipec8Eeeu0xXdbba9frFj0=OqFfea0dXdd9vqai=hGuQ8kuc9pgc9s8qqaq=dirpe0xb9q8qiLsFr0=vr0=vr0dc8meaabaqaciaacaGaaeqabaqabeGadaaakeaadaaeabqaaiqbdsha0zaaDaWaaSbaaSqaaiabdIgaObqabaGccuWGWbaCgaqhamaaDaaaleaacqWGObaAaeaacqWGubavaaaabeqab0GaeyyeIuoaaaa@361D@ + **E **    (9)

**Y **= **U Q**^*T *^+ **F **= ∑u¯hq¯hT
 MathType@MTEF@5@5@+=feaafiart1ev1aaatCvAUfKttLearuWrP9MDH5MBPbIqV92AaeXatLxBI9gBaebbnrfifHhDYfgasaacH8akY=wiFfYdH8Gipec8Eeeu0xXdbba9frFj0=OqFfea0dXdd9vqai=hGuQ8kuc9pgc9s8qqaq=dirpe0xb9q8qiLsFr0=vr0=vr0dc8meaabaqaciaacaGaaeqabaqabeGadaaakeaadaaeabqaaiqbdwha1zaaDaWaaSbaaSqaaiabdIgaObqabaGccuWGXbqCgaqhamaaDaaaleaacqWGObaAaeaacqWGubavaaaabeqab0GaeyyeIuoaaaa@3621@ + **F **    (10)

Because it is possible to let the matrices **T **and **U **(referred to as the "score" matrices) represent the variable matrices **X **and **Y**, a mixed inner relation can be established using:

**Y **= **T B Q**^*T *^+ **E **    (11)

**Figure 4:**
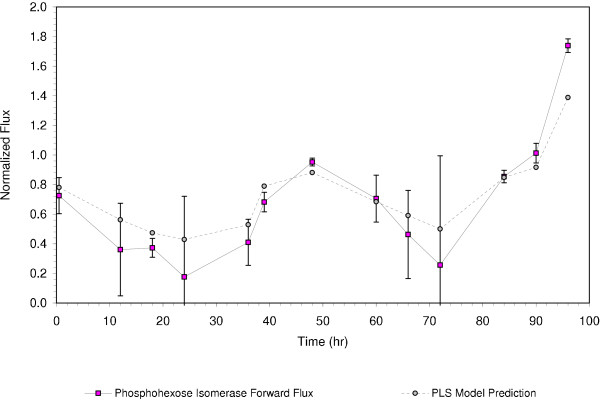
Partial Least Squares model prediction of glycolytic flux based upon gene transcription values.

The resulting model is shown below in Figure 4.

In Figure 4, the PLS model prediction based upon gene transcription data correlates with the flux measurements. The resulting model selected 132 of the 3,185 genes in the study to predict the glycolytic flux. Indeed, when the model was recreated using random sets of genes, none of the random gene sets predicted the data as well as our model, nor did any of the resulting models capture as much of the variance as our model [9].

Although PLS is a powerful correlation algorithm for linking different types of multivariate data, care must be used in its application. Because gene transcription data sets often contain many more genes than samples, we conducted a number of studies using random data to determine if statistically significant models could be derived between unrelated data sets [9]. It was found that if the number of genes is much greater than the number of samples, accurate model predictions could arise by chance from random data. Thus to have relevant models, the number of samples used must make the data matrix closer to full rank than is typical in most microarray experiments. For full genome arrays this would require thousands of samples, which is prohibitive in most experiments. Given these circumstances, PLS may not be a suitable tool for discovering new relationships between gene transcription data and other biochemical data contained within the Y-block. This does not preclude the useful application of PLS to either discovery, or for modeling biological systems where full rank data may be obtained. It does necessitate careful planning in the prudent use of the technique.

Although there are usually many more genes than samples in microarray experiments, depending upon the experiment there may be effective ways to limit the gene domain. Most of these rely upon either rigorous computational selection methods (for example, tests for reliable signals or differential expression), or biological hypotheses that can be used to study a sub-set of the genes with respect to the desired outputs (in which case measuring transcript levels using RT-PCR may provide more accurate data). In these cases the researcher is either assuming that most of the relevant genes are in the model, or statistically tests the gene set to try and find a relevant subset.

Another systems identification algorithm is called Time Lagged Correlations (TLC) [63]. TLC is based upon clustering, and therefore can incorporate any data type, however, it goes beyond identifying simple relationships to identifying directional relationships.

The various forms of clustering [43,47,65] employed to date have produced potential gene relationships and in some cases have yielded the identity of transcription factor binding motifs. Despite their success, these methods are limited in their ability to infer causality or directional relationships between genes and other data types. The results of clustering algorithms yield relations such as "transcription of gene *A *predicts transcription of gene *B*," which is the same as saying "transcription of gene *B *predicts transcription of gene *A*." Neither Bayesian networks [66], nor information theory based approaches [67] have made use of the sequential nature of time-series data in current applications. When enough time points are available to prevent over fitting the data and find statistically significant correlations, a discovery method to uncover potential causal relationships among genes and other data types may be attempted. Directionality is incorporated into probabilistic networks by determining the temporal order in which expression patterns are affected in a sequence.

Transcriptional regulatory behavior can be examined by probing the *dynamics *of gene expression in carefully designed experiments covering a wide range of conditions. Dynamic experiments that sequentially vary external parameters (such as diet composition, amount, or energy expenditure) offer insights into how cellular physiology depends on changing environmental conditions. TLC analysis can be used to identify putative causal relationships between system perturbations and responses. TLC uses linear Pearson Correlations [27] by determining the best correlations between transcript profiles shifted in time. For a transcription profile representing *n *measurements taken at equally spaced time points, the correlation between genes *i *and *j *with a time lag, *τ*, is **R**(*τ*) = (r_*ij*_(*τ*)), defined as

*S*_*ij*_(*τ*) = ⟨(*x*_*i*_(*t*) - x¯i
 MathType@MTEF@5@5@+=feaafiart1ev1aaatCvAUfKttLearuWrP9MDH5MBPbIqV92AaeXatLxBI9gBaebbnrfifHhDYfgasaacH8akY=wiFfYdH8Gipec8Eeeu0xXdbba9frFj0=OqFfea0dXdd9vqai=hGuQ8kuc9pgc9s8qqaq=dirpe0xb9q8qiLsFr0=vr0=vr0dc8meaabaqaciaacaGaaeqabaqabeGadaaakeaacuWG4baEgaqeamaaBaaaleaacqWGPbqAaeqaaaaa@2FC4@)(*x*_*j *_(*t *+ *τ*) - x¯j
 MathType@MTEF@5@5@+=feaafiart1ev1aaatCvAUfKttLearuWrP9MDH5MBPbIqV92AaeXatLxBI9gBaebbnrfifHhDYfgasaacH8akY=wiFfYdH8Gipec8Eeeu0xXdbba9frFj0=OqFfea0dXdd9vqai=hGuQ8kuc9pgc9s8qqaq=dirpe0xb9q8qiLsFr0=vr0=vr0dc8meaabaqaciaacaGaaeqabaqabeGadaaakeaacuWG4baEgaqeamaaBaaaleaacqWGQbGAaeqaaaaa@2FC6@)⟩     (12)

rij(τ)=Sij(τ)Sii(τ)Sjj(τ)     (13)
 MathType@MTEF@5@5@+=feaafiart1ev1aaatCvAUfKttLearuWrP9MDH5MBPbIqV92AaeXatLxBI9gBaebbnrfifHhDYfgasaacH8akY=wiFfYdH8Gipec8Eeeu0xXdbba9frFj0=OqFfea0dXdd9vqai=hGuQ8kuc9pgc9s8qqaq=dirpe0xb9q8qiLsFr0=vr0=vr0dc8meaabaqaciaacaGaaeqabaqabeGadaaakeaacqWGYbGCdaWgaaWcbaGaemyAaKMaemOAaOgabeaakiabcIcaOGGaciab=r8a0jabcMcaPiabg2da9maalaaabaGaem4uam1aaSbaaSqaaiabdMgaPjabdQgaQbqabaGccqGGOaakcqWFepaDcqGGPaqkaeaadaGcaaqaaiabdofatnaaBaaaleaacqWGPbqAcqWGPbqAaeqaaOGaeiikaGIae8hXdqNaeiykaKIaem4uam1aaSbaaSqaaiabdQgaQjabdQgaQbqabaGccqGGOaakcqWFepaDcqGGPaqkaSqabaaaaOGaaCzcaiaaxMaadaqadaqaaiabigdaXiabiodaZaGaayjkaiaawMcaaaaa@511E@

where x_*i*_(*t*) is the expression of gene *i *at time *t*, x¯i
 MathType@MTEF@5@5@+=feaafiart1ev1aaatCvAUfKttLearuWrP9MDH5MBPbIqV92AaeXatLxBI9gBaebbnrfifHhDYfgasaacH8akY=wiFfYdH8Gipec8Eeeu0xXdbba9frFj0=OqFfea0dXdd9vqai=hGuQ8kuc9pgc9s8qqaq=dirpe0xb9q8qiLsFr0=vr0=vr0dc8meaabaqaciaacaGaaeqabaqabeGadaaakeaacuWG4baEgaqeamaaBaaaleaacqWGPbqAaeqaaaaa@2FC4@ is the average expression value of gene *i *across all time points, and the angled brackets represent the inner product between the time-shifted profiles [63]. The matrix of lagged correlations **R**(*τ*) can be used to rank the correlation and anticorrelation between genes through conversion to a Euclidean distance metric, d_*ij*_:

*d*_*ij *_= (*c*_*ij *_- 2*c*_*ij *_+ *c*_*jj*_)^1/2 ^= 2
 MathType@MTEF@5@5@+=feaafiart1ev1aaatCvAUfKttLearuWrP9MDH5MBPbIqV92AaeXatLxBI9gBaebbnrfifHhDYfgasaacH8akY=wiFfYdH8Gipec8Eeeu0xXdbba9frFj0=OqFfea0dXdd9vqai=hGuQ8kuc9pgc9s8qqaq=dirpe0xb9q8qiLsFr0=vr0=vr0dc8meaabaqaciaacaGaaeqabaqabeGadaaakeaadaGcaaqaaiabikdaYaWcbeaaaaa@2DB9@(1.0 - *c*_*ij*_)^1/2 ^    (14)

*c*_*ij *_= *max*|*r*_*ij*_(*τ*)|     (15)

where, c_*ij *_is the maximum absolute value of the correlation between two genes at a time lag *τ*. If the value of *τ *that gives the maximum correlation is zero, then the two genes are best correlated with no time lag. The matrix **D **= (d_*ij*_) describes the correlation between two genes, *i *and *j*, in terms of "distance" by making genes that are least correlated (for any *τ*) the "farthest" apart [68]. Thus transforming the correlation matrix, **R**, into a distance matrix, **D**, allows anti-correlated genes to be included in the network, in addition to correlated genes. By finding genes that are closely related and then examining the corresponding value of *τ*, an underlying network of potential cause and effect relationships can be assembled. Some caution is needed to ensure genes with high correlation have been chosen using enough data points to give statistical significance, otherwise all of the *τ *values used will overfit the data. Such errors may be obvious if values for *τ *are unreasonably long from a biological standpoint.

**Figure 5:**
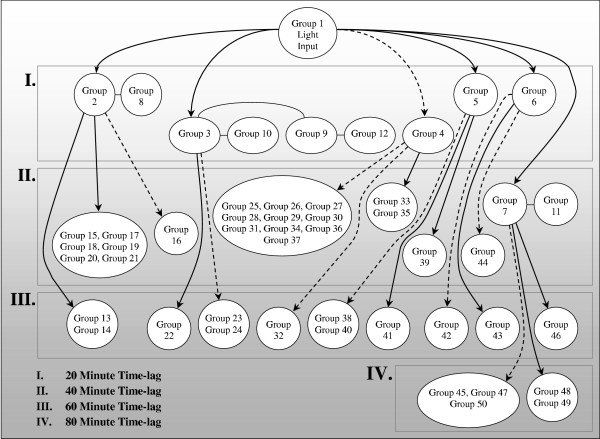
Gene interaction network derived from time lagged correlation analysis using gene transcription data. Solid lines represent gene groups with correlation at the indicated time lag, while broken lines represent gene groups that are anticorrelated. The final network comprises 50 gene groups containing 259 genes.

To demonstrate the application of TLC to transcriptional data, we studied metabolism in the photosynthetic bacterium, *Synechocystis *sp., that was exposed to different light conditions [63]. Dynamic light perturbations were induced to drive the transcriptional changes in the bacteria, which were measured using DNA microarrays. The gene transcription responses were then placed into a network based upon their time lagged correlations to either the input light signal or another gene cluster, providing a set of putative causal relationships that could be subsequently test. After collecting transcriptional data from over 47 time points, the network shown in Figure 5 was constructed.

As other measurements such as protein and metabolite data become available, TLC studies should allow for the creation of hypothetical networks similar to that in Figure 5, but with greater degrees of mechanistic information. Such approaches will hold new insights into the regulation of energy homeostasis by linking various data sets in maps that show putative directional connections.

### Integrating complementary data types to study energy homeostasis

Once a transcriptional study has been conducted and important genes have been identified, further verification of the genetic contributions to the underlying phenotype is necessary. The type of studies that are included depend largely on the experimental hypothesis, phenotype under investigation, and model system. Many of these verification studies may at least begin *in silico *by additional analysis of the identified genes across a number of databases [69]. Today databases are available that list common biological pathways in which the gene product may participate, mutations, single nucleotide polymorphisms (SNPs), and mutant animals that are available for many genes. In addition, a number of specific resources exist, particularly for phenotypes related to energy homeostasis [3].

Despite the experiment-specific nature of continued studies, a few tools have recently been developed that contribute to a systematic approach for additional gene verification studies. These include the use of RNA interference (RNAi), synthetic gene construction, and analysis techniques that link mutations to phenotypes.

One of the most effective ways to investigate how a gene influences a phenotype is to disrupt or eliminate the gene product and then observe changes in the phenotype. Although *in vivo *manipulation of genes can be very time consuming and high-through put evaluation is currently prohibitive for most laboratories, RNAi has been effectively used to silence genes and generate "functional" gene knock-outs in cellular models [2,70] and whole animals [71-73]. RNAi can therefore be used to screen loss of function gene effects on phenotypes of interest.

RNAi works by transfecting cells with double stranded RNAs. Delivery of the RNA may be transient, relying on direct transfection of synthesized RNAs, or stable, by transfecting viral vectors that expression double stranded RNAs [74]. Once inside the cell, the double stranded RNA activates a protein catalyzed pathway through which specific natively transcribed RNAs are degraded or not translated [75]. RNAi has already been employed in numerous investigations that study energy homeostasis [76-79]. When combined with microarray experiments, RNAi can be used to rapidly screen individual or groups of genes that are identified in the analysis. For example, the gene network described in Figure 5 postulates a number of activation (solid lines in the figures) and repressive relationships (dotted lines in the figures). Genes that reside in group 5, which are proposed to activate genes in groups 39 and 41, can be silenced iteratively in subsequent experiments and the network reconfigured to see if gene membership in the network remains the same and whether the genes in groups 39 and 41 are not activated in response to silencing of genes in group 5 (as is proposed by the figure).

**Figure 6:**
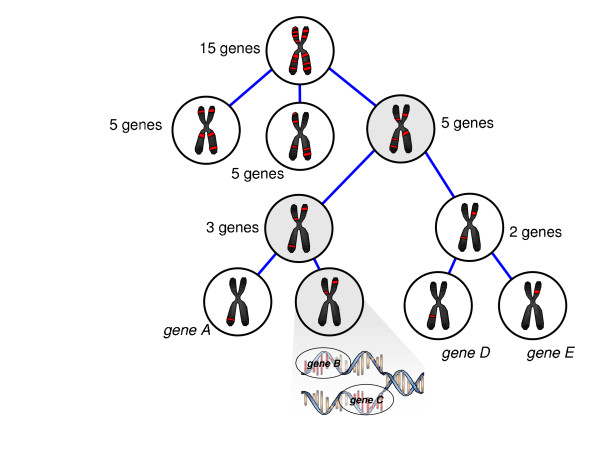
Combinatorial siRNA screening strategy for 15 genes. Using this approach the primary effects of a single gene could be discovered using a limited number of experiments. In this case cells were exposed to groups of siRNAs, as opposed to individual sequences. In this example, siRNA groups highlighted in gray have the primary effect on the phenotype, which can be mapped down to the single gene, either *gene B *or *gene C*, using a binary search.

Experiments using RNAi based screening can be conducted in a high-through put manner in cellular systems and some more complex experimental models [80,81]. In our laboratory we rapidly screened 15 overexpressed genes for their effects on hepatic insulin resistance using a combinatorial approach in which genes are silenced simultaneously as described in Figure 6. Using this strategy we were able to identify three genes that had an effect on hepatic glucose output in primary cells using seven experiments as opposed to 15 (unpublished data). While a powerful tool for rapidly finding relevant genes, it must be practiced with some care: interactions that occur from silencing genes simultaneously may be hidden and difficult to detect. Despite the drawbacks, this approach sacrifices detailed observations on all individual effects for rapid screening, which is often preferred if the gene set under investigation is large.

Similarly, modern gene synthesis provides a complementary approach to gene silencing by enabling overexpression studies. Again referring to Figure 5, repression of group 4 genes appears to subsequently activate group 33 and group 35 genes. In this case the relationship could be tested by synthesizing expression vectors for the genes within group 4 and seeing if their overexpression leads to continued repression of genes in groups 33 and 35 under the same experimental conditions.

Gene synthesis has a number of other useful applications. Another strategy for investigating cellular network architecture would be to overexpress transcription factors that are hypothesized to drive transcription of an identified cluster of genes. In this type of experiment, one would look to see which genes in the cluster were up-regulated in the presence of transcription factor overexpression. Additionally, gene synthesis could be used to study the effects of various mutants, and characterize recombinant proteins *in vitro *to determine how mutations affect their biochemistry. Because the cost of gene synthesis has rapidly decreased in recent years [82,83], these types of experiments are becoming more amenable for most laboratories to perform.

Finally, in the 1990's it became possible to systematically map quantitative trait loci (QTLs) and over 2,000 different QTLs have been identified in a range of rodent phenotypes including obesity [84,85] and diabetes [86]. Despite the improving feasibility of association studies [4], linkage studies [5], admixture studies and others that can identify QTLs, less than 1% of these QTLs have been characterized at the molecular level [87]; that is, an important region of the genome has been identified, but the actual gene(s) or genetic element (s) contributing to the QTL remain unknown.

The value of QTL analysis to discovering disease genes is in reducing the region of the genome under investigation. Once this has been done, other techniques such as DNA sequencing, array based SNP identification, positional cloning, and transgenic knockouts can be used to search for genes within the identified locus. By the end of 2001, this approach had resulted in the discovery of 29 disease genes, eight of which were involved in diabetes or obesity [88]. Genes discovered through QTL analysis are often highly penetrant (Penetrance is the number of individuals within a population that have a specific genotype and the corresponding phenotype), with a large effect size (Effect size is the amount, or percentage, of phenotypic variation that is attributable to a QTL). This is a major drawback to finding all relevant genes to a particular phenotype through QTL analysis alone. QTL analysis requires time consuming experiments and a large number of samples: 1,000 animals will only map a QTL contributing 5% of the phenotype variation onto a 10 centimorgan (cM) interval with 50% power [89]. Because it is a mapping technique, the gene or genetic element must still be identified, which can be challenging particularly if the element is relatively small (with low information content) or resides is a region with many polymorphisms.

Combining QTL analysis with DNA microarray results is a complementary approach that has already resulted in the identification of two disease-related genes [88], one of which is involved in insulin-mediated glucose uptake in rats [90]. Cross-referencing genes identified in microarray experiments with genomic regions identified in QTL studies may help single out specific genes for more detailed work. Considering DNA microarray analysis does not necessarily require 100's of samples, combining these results with QTL analysis and other techniques such as multiple linear regression [91], which relate genomic regions or SNPs to phenotypes of interest, promises to further our understanding of the genetic regulation of energy homeostasis.

## Conclusion

Effectively employing genome scale technologies, such as DNA microarrays, has thus far provided unique challenges in experimental design, data analysis, and data integration. Many of these problems are particularly challenging for clinical researchers who would like to incorporate larger amounts of molecular data into their investigations, but have not previously dealt with multivariate problems at a similar scale. DNA microarrays, when used in carefully designed experiments can enable systems identification and gene discovery, which is critical to defining the molecular basis of energy homeostasis. Combining this technology with other complementary methods of analysis and experimental tools should help define the most relevant molecular pathways and holds the promise of providing new clinical insights.

## Competing interests

The author(s) declare that they have no competing interests.

## Authors' contributions

RMR wrote this manuscript.
